# Evaluation of Alterations in Serum Uric Acid Levels in Newly Diagnosed Overt Thyroid Dysfunction: A Prospective Observational Study From a Tertiary Care Center

**DOI:** 10.7759/cureus.116248

**Published:** 2026-09-14

**Authors:** Deepika T, Yatin Nigam, Suyasha Choudhary, Adarsh Krishna K Bhat

**Affiliations:** 1 Internal Medicine, Rajarajeswari Medical College and Hospital, Bengaluru, IND; 2 Orthopaedic Surgery, Gleneagles BGS Hospital, Bengaluru, IND

**Keywords:** hyperthyroidism, hypothyroidism, metabolic profile, serum uric acid, thyroid dysfunction

## Abstract

Background: Thyroid dysfunction is widely recognized to affect metabolic regulation and renal handling of uric acid and creatinine, but historical clinical findings have remained inconsistent. This study evaluates biochemical profiles and diagnostic markers across control, hyperthyroid, and hypothyroid cohorts to establish clear correlations and diagnostic thresholds.

Objectives: To comprehensively analyze baseline demographic characteristics, thyroid profiles (T3, T4, TSH (thyroid stimulating hormone)), and renal parameters (uric acid, creatinine) across patient cohorts; to explore Pearson correlations among these metrics; and to conduct receiver operating characteristic (ROC) curve analysis to determine the diagnostic utility and optimal cutoffs for serum uric acid and creatinine as biomarkers for thyroid dysfunction.

Methods: A comprehensive cross-sectional analysis was performed on a study population of N = 300 participants, split into a control group (n = 150) and a thyroid dysfunction group (n = 150, comprising 50 hyperthyroid and 100 hypothyroid patients) at a tertiary care center over 12 months. Detailed biochemical profiling, Pearson correlation coefficients, and ROC curve analyses with sensitivity/specificity calculations were performed to assess marker efficacy.

Results: The overall mean age was 33.10 ± 13.65 years, with a balanced distribution across cohorts (p = 0.137). Significant discrepancies in T3 (p = 0.004) and TSH (p < 0.001) were confirmed. Serum uric acid was significantly elevated in thyroid dysfunction compared with controls (5.62 ± 2.28 vs. 4.46 ± 1.13 mg/dL, p < 0.001), showing a strong positive correlation with TSH (r = 0.197, p = 0.001). Conversely, serum creatinine was modestly but significantly lower in the thyroid dysfunction group (0.86 ± 0.16 vs. 0.92 ± 0.22 mg/dL, p = 0.010), showing a significant negative correlation with TSH (r = -0.176, p = 0.002). ROC analysis revealed significant diagnostic accuracy for uric acid (area under the curve (AUC) = 0.642, p < 0.001), with an optimal screening cutoff of 4.05 mg/dL (71.3% sensitivity), whereas creatinine demonstrated poor discriminatory performance (AUC = 0.441, p = 0.076).

Conclusion: Serum uric acid demonstrates highly significant alterations and predictable diagnostic value in thyroid dysfunction, closely correlating with TSH levels. Creatinine shows a reverse metabolic association and poor diagnostic accuracy. Routine screening of serum uric acid, but not necessarily creatinine, is clinically indicated in newly diagnosed thyroid dysfunction cases to guide metabolic risk stratification.

## Introduction

Thyroid diseases are among the most common endocrine disorders worldwide, affecting nearly 42 million people in India [1]. Increased awareness and access to laboratory testing have significantly improved the detection of primary thyroid disorders. The thyroid gland secretes thyroxine (T4) and triiodothyronine (T3), which influence cellular metabolism, thermoregulation, and developmental processes. Autoimmune mechanisms may result in either excessive hormone production (hyperthyroidism) or reduced function (hypothyroidism).

Thyroid hormone imbalance is known to affect multiple physiological systems, including lipid metabolism, cardiovascular function, and renal physiology [2-4]. Individuals with overt thyroid disorders often present with dyslipidemia, left ventricular diastolic dysfunction, reduced estimated glomerular filtration rate (eGFR), and increased cardiovascular risk, many of which are reversible with appropriate treatment.

Uric acid is the final metabolite of purine breakdown, and elevated serum uric acid increases the risk of gout and metabolic comorbidities such as hypertension, chronic kidney disease, metabolic syndrome, and diabetes mellitus [5-7]. Given potential overlapping metabolic pathways, researchers have explored whether thyroid dysfunction contributes to changes in uric acid metabolism.

Some studies have observed altered uric acid levels in hypothyroidism, linked to reduced renal perfusion and slower excretion [8,9], while others found no consistent associations. The National Health and Nutrition Examination Survey reported that elevated serum uric acid independently increases cardiovascular mortality, similar to thyroid dysfunction [10]. However, local data exploring this relationship are limited.

Although previous studies have attempted to establish an association between hyperuricemia and thyroid disorders, the findings have been inconsistent and contradictory. The lack of consensus regarding this relationship, along with the limited availability of regional data, highlights the need for further evaluation of serum uric acid patterns in patients with overt thyroid dysfunction. Studying the association between thyroid dysfunction and hyperuricemia may help clarify its clinical significance and determine whether thyroid status influences serum uric acid levels.

The present study aims to assess the risk of hyperuricemia among individuals newly diagnosed with overt hypothyroidism and hyperthyroidism. It also aims to determine whether routine screening of serum uric acid levels is warranted in patients with thyroid dysfunction.

## Materials and methods

This study was designed as a prospective cross-sectional observational analysis conducted over a duration of 12 months from August 1, 2024, to July 31, 2025, at the Department of General Medicine, Rajarajeswari Medical College and Hospital, Bengaluru. Approval from the Institutional Ethics Committee, Rajarajeswari Medical College and Hospital, Bengaluru, India, was sought on July 23, 2024. The study population included patients attending both outpatient and inpatient services who were newly diagnosed with overt thyroid dysfunction based on standard biochemical criteria.

Ethical approval for the study was obtained from the Institutional Ethics Committee prior to commencement. All procedures followed were in accordance with the ethical standards of the institutional research committee and adhered to the principles outlined in the Declaration of Helsinki. Written informed consent was obtained from all participants before enrollment in the study.

Inclusion criteria

The study included adults aged 18 years and above who were newly diagnosed with overt hypothyroidism or hyperthyroidism. Only individuals who had not received any prior treatment for thyroid dysfunction were included in the study.

Exclusion criteria

Individuals with known kidney disease were excluded from the study. Patients on chronic treatment with diuretics or urate-lowering medications were also excluded, as these medications may influence serum uric acid levels. Pregnant women were likewise excluded from participation.

The final study cohort encompassed an aggregate population of N = 300 adults, including 150 healthy adults and 150 patients with newly diagnosed overt thyroid dysfunction.

Sample size calculation

The sample size was calculated based on the expected prevalence of hyperuricemia among patients with thyroid dysfunction, using the standard formula for estimation of a single proportion:

$n = \frac{Z&sup2;pq}{d&sup2;}$

where n is the required sample size, Z is the standard normal deviate corresponding to a 95% confidence level (1.96), p is the anticipated prevalence of hyperuricemia, q = 1 − p, and d is the allowable absolute precision. Based on the assumed prevalence and precision, the minimum required sample size was calculated, and an adequate number of participants was subsequently enrolled. A total of 150 patients with newly diagnosed overt hypothyroidism or hyperthyroidism who fulfilled the predefined inclusion and exclusion criteria were included in the final analysis. An additional 150 apparently healthy adults were recruited as a comparison group, resulting in a total study population of 300 participants.

Data collection and analysis

Demographic information, thyroid function parameters, serum uric acid levels, and renal function tests were recorded. Data entry was performed using Microsoft Excel (Microsoft Corporation, Redmond, Washington) and analyzed using IBM SPSS Statistics for Windows, Version 22 (Released 2013; IBM Corp., Armonk, New York). Categorical variables were presented as frequencies and percentages. Chi-square test or Fisher’s exact test was used for comparison of proportions where appropriate. A p-value of <0.05 was considered statistically significant.

## Results

A total of 300 participants were fully evaluated and split into a healthy control group (n = 150), an overt hyperthyroid group (n = 50), and an overt hypothyroid group (n = 100). The full comparative spectrum of baseline demographic features, thyroid hormone concentrations, and renal biomarkers across these three primary groups is provided in Table 1.

**Table 1:** Baseline Demographic and Biochemical Parameters Across Study Groups Data are presented as mean ± standard deviation (SD). Comparisons among the control, hyperthyroid, and hypothyroid groups were performed using one-way analysis of variance (ANOVA). A p-value < 0.05 was considered statistically significant. TSH: thyroid-stimulating hormone.

Variable	Control (n = 150)	Hyperthyroid (n = 50)	Hypothyroid (n = 100)	Overall (N = 300)	p-value
Age (years)	32.13 ± 7.87	36.54 ± 28.27	32.84 ± 8.14	33.10 ± 13.65	0.137
T3	1.01 ± 0.32	0.92 ± 0.51	0.84 ± 0.40	0.94 ± 0.39	0.004
T4	7.99 ± 1.71	0.79 ± 0.84	12.39 ± 74.53	8.26 ± 43.08	0.297
TSH	2.20 ± 1.02	0.74 ± 0.68	8.01 ± 9.92	3.89 ± 6.48	<0.001
Uric acid (mg/dL)	4.46 ± 1.13	5.59 ± 2.17	5.63 ± 2.35	5.04 ± 1.89	<0.001
Creatinine (mg/dL)	0.92 ± 0.22	0.87 ± 0.16	0.85 ± 0.16	0.89 ± 0.19	0.035

The mean age of the entire cohort was 33.10 ± 13.65 years, showing strong statistical similarity and demographic comparability across all groups (p = 0.137). Circulating T3 levels differed significantly across groups (p = 0.004), showing marked suppression in hypothyroid patients. Serum TSH displayed an exceptionally robust variation (p < 0.001), exhibiting severe suppression in the hyperthyroid cohort and a striking elevation in hypothyroid subjects. Crucially, renal indices also demonstrated significant modifications; serum uric acid values peaked in both hyperthyroid and hypothyroid cohorts relative to controls (p < 0.001), while creatinine levels revealed a modest but statistically significant decline in the disease groups (p = 0.035).

To determine the exact continuous interaction between circulating hormones and renal handling, a Pearson linear correlation model was computed across the overall population (Table 2). 

**Table 2:** Pearson Correlation Profiles Between Thyroid Hormones and Renal Markers Pearson correlation coefficients (r) were used to assess the relationship between thyroid hormone levels and serum uric acid and creatinine. A p-value < 0.05 was considered statistically significant. TSH: thyroid-stimulating hormone.

Variables Compared	Pearson’s r Coefficient	p-value
T3 vs uric acid	−0.087	0.133
T3 vs creatinine	0.001	0.992
T4 vs uric acid	0.129	0.026
T4 vs creatinine	0.018	0.751
TSH vs uric acid	0.197	0.001
TSH vs creatinine	−0.176	0.002

The analysis revealed that TSH shares a highly significant positive correlation with serum uric acid (r = 0.197, p = 0.001), confirming a progressive rise in uric acid as thyroid function declines. Conversely, TSH maintains a significant negative correlation with serum creatinine (r = −0.176, p = 0.002). T4 displayed a weak but statistically significant positive correlation with uric acid (r = 0.129, p = 0.026), whereas T3 demonstrated no statistical relationship with either metric.

Gender distribution metrics remained stable and balanced across all three categorical groups, as illustrated in Table 3. Females uniformly represented approximately two-thirds of the overall study population, with no statistically significant association between patient sex and final thyroid status diagnosis (p = 0.482).

**Table 3:** Gender Distribution Patterns Across Diagnosed Groups Data are presented as frequencies and percentages. The association between gender and thyroid function group was assessed using the chi-square test. A p-value < 0.05 was considered statistically significant.

Gender	Control (n = 150)	Hyperthyroid (n = 50)	Hypothyroid (n = 100)	Total (N = 300)	p-value
Male	49 (32.7%)	21 (42.0%)	36 (36.0%)	106 (35.3%)	0.482
Female	101 (67.3%)	29 (58.0%)	64 (64.0%)	194 (64.7%)	

Consolidated group assessment: controls vs. total thyroid dysfunction

To look at macro-level metabolic shifts, the hyperthyroid and hypothyroid groups were pooled into a unified “Thyroid Dysfunction Group” (n = 150) and directly evaluated against the healthy control group (n = 150), as summarized in Table 4.

**Table 4:** Comparison of Demographic, Thyroid, and Renal Parameters Between Groups (N = 300) Data are presented as mean ± standard deviation (SD). Comparisons between the control and thyroid dysfunction groups were performed using one-way analysis of variance (ANOVA). A p-value <0.05 was considered statistically significant.

Variable	Control (n = 150)	Thyroid Group (n = 150)	Overall (N = 300)	p-value
Age (years)	32.13 ± 7.87	34.07 ± 17.61	33.10 ± 13.65	0.217
T3	1.01 ± 0.32	0.87 ± 0.44	0.94 ± 0.39	0.002
T4	7.99 ± 1.71	8.52 ± 61.00	8.26 ± 43.08	0.914
TSH	2.20 ± 1.02	5.59 ± 8.80	3.89 ± 6.48	<0.001
Uric acid (mg/dL)	4.46 ± 1.13	5.62 ± 2.28	5.04 ± 1.89	<0.001
Creatinine (mg/dL)	0.92 ± 0.22	0.86 ± 0.16	0.89 ± 0.19	0.010

This macro-level assessment confirms that serum uric acid levels are significantly higher in the consolidated thyroid dysfunction group compared with healthy controls (5.62 ± 2.28 vs. 4.46 ± 1.13 mg/dL, p < 0.001). Conversely, serum creatinine was modestly but significantly lower in the thyroid dysfunction group (0.86 ± 0.16 vs. 0.92 ± 0.22 mg/dL, p = 0.010).

Receiver operating characteristic (ROC) predictive modeling

To determine the diagnostic efficacy of these renal metrics as active biomarkers for predicting underlying thyroid dysfunction, a formal ROC analysis was executed (Table 5 and Figure 1). 

**Table 5:** ROC Curve Analysis for Uric Acid and Creatinine in Predicting Thyroid Dysfunction Receiver operating characteristic (ROC) curve analysis was performed to evaluate the discriminatory ability of serum uric acid and creatinine for identifying thyroid dysfunction. The area under the curve (AUC) with a 95% confidence interval (CI) was calculated. A p-value <0.05 was considered statistically significant.

Test Variable	AUC (ROC)	Std. Error	95% Confidence Interval	p-value
Uric acid	0.642	0.032	0.578-0.705	<0.001
Creatinine	0.441	0.033	0.376-0.506	0.076

**Figure 1:**
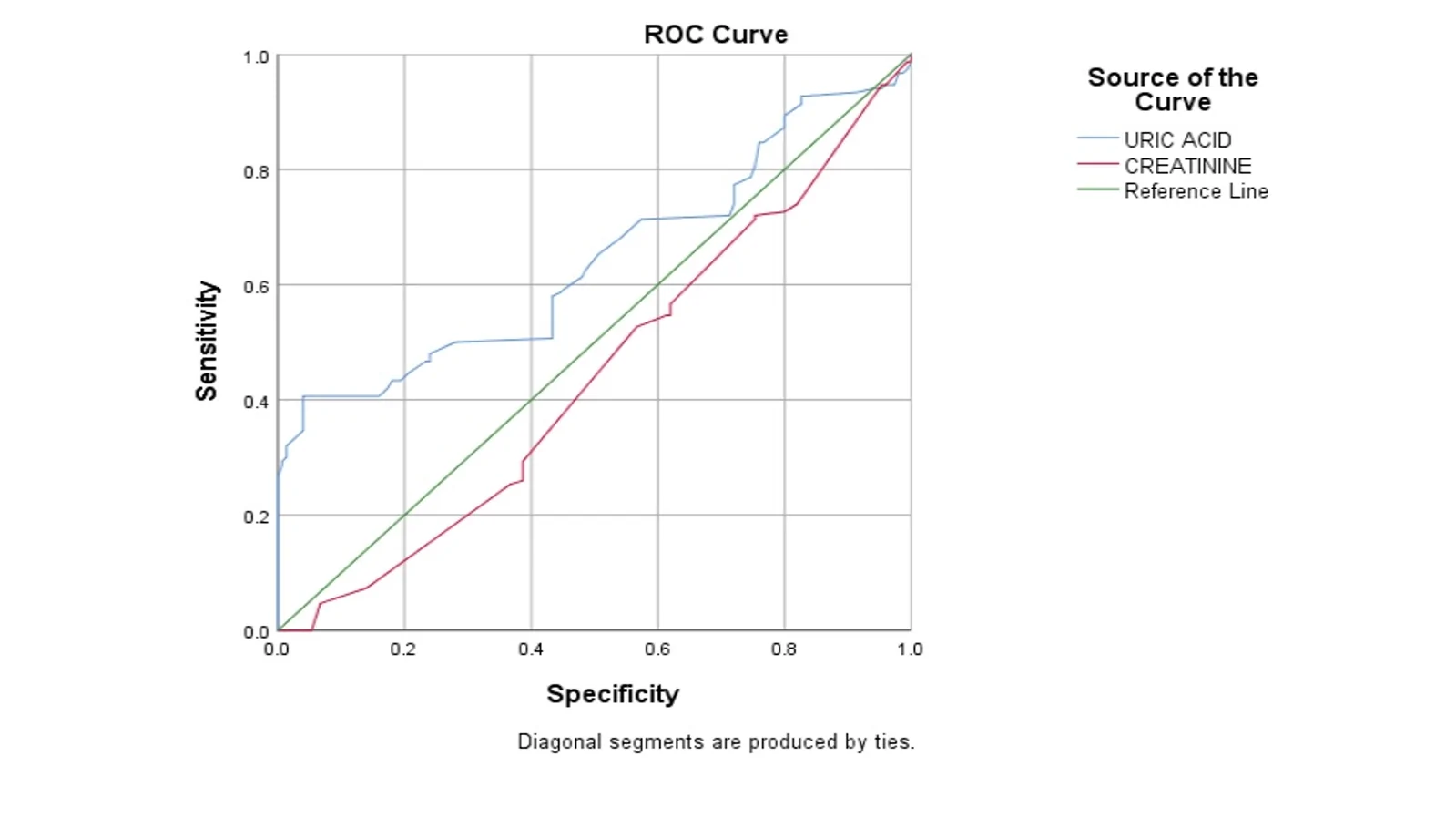
Receiver Operating Characteristic (ROC) Curve

Serum uric acid possesses statistically significant diagnostic accuracy in distinguishing thyroid dysfunction from a healthy baseline, yielding an AUC of 0.642 (95% CI: 0.578-0.705, p < 0.001). Conversely, serum creatinine exhibits an AUC of 0.441 (95% CI: 0.376-0.506, p = 0.076), confirming that its discriminatory capacity is no better than random chance. Table 6 and Table 7 outline the sensitivity and specificity values across different diagnostic cutoffs. 

**Table 6:** Optimal Diagnostic Cutoff and Performance Coordinates for Serum Uric Acid Sensitivity and specificity were calculated for different serum uric acid cutoff values for discrimination between participants with thyroid dysfunction and controls.

Cutoff Value (≥ mg/dL)	Sensitivity	Specificity
4.05 mg/dL	71.3%	42.7%
5.05 mg/dL	50.0%	72.0%

**Table 7:** Diagnostic Performance Matrix of Serum Creatinine Cutoff Coordinates Sensitivity and specificity were calculated for different serum creatinine cutoff values for discrimination between participants with thyroid dysfunction and controls.

Cutoff Value (≥ mg/dL)	Sensitivity	Specificity
0.73 mg/dL	74.0%	18.0%
0.84 mg/dL	56.7%	38.0%
0.92 mg/dL	29.3%	61.3%
1.05 mg/dL	7.3%	86.0%

## Discussion

The empirical outcomes derived from this study establish a clear, statistically validated relationship between overt thyroid dysfunction and alterations in renal/metabolic indices, resolving historical discrepancies. Crucially, our data demonstrate that serum uric acid levels undergo highly significant modifications during thyroid dysfunction, exhibiting substantial elevation in both hyperthyroid (5.59 ± 2.17 mg/dL) and hypothyroid (5.63 ± 2.35 mg/dL) cohorts relative to healthy baselines (4.46 ± 1.13 mg/dL, p < 0.001). This starkly contradicts previous ambiguous or localized assumptions suggesting that hyperuricemia and thyroid pathology are entirely independent or random coexisting events [11,12].

The biological mechanism driving these findings is supported by our Pearson correlation analysis, which demonstrates a robust positive correlation between TSH and serum uric acid (r = 0.197, p = 0.001). In hypothyroid states, the significant rise in uric acid levels can be attributed to decreased renal blood flow, reduced glomerular filtration, and altered tubular secretion of urate [3,8,9]. In hyperthyroid states, accelerated purine metabolic turnover and a heightened oxidative state prompt a comparable increase in circulating uric acid, overriding concurrent increases in renal clearance [13]. This dual-pathway elevation explains why serum uric acid acts as a meaningful metabolic indicator across thyroid disorders.

Furthermore, our macro-level aggregated group comparison (Table 4) solidifies this link, showing a definitive surge in uric acid within the consolidated dysfunction group (5.62 ± 2.28 mg/dL) versus the control group (p < 0.001). This relationship is further validated by ROC curve modeling, where serum uric acid achieved a statistically significant AUC of 0.642 (p < 0.001). By examining specific cutoffs, a value of ≥4.05 mg/dL yields a high sensitivity of 71.3%, marking it as a strong clinical screening threshold for detecting underlying metabolic strain in thyroid patients, while a higher threshold of ≥5.05 mg/dL offers enhanced specificity (72.0%) for risk stratification [5-7,10].

Conversely, serum creatinine behaves in an entirely separate, opposing manner. Aggregated analysis shows that creatinine is modestly but significantly lower in patients with thyroid dysfunction compared with healthy individuals (0.86 ± 0.16 vs. 0.92 ± 0.22 mg/dL, p = 0.010), maintaining a significant negative correlation with circulating TSH (r = −0.176, p = 0.002). This negative shift reflects changes in skeletal muscle turnover and altered creatinine clearance pathways typical of active thyroid pathology. However, due to its poor ROC performance (AUC = 0.441, p = 0.076), serum creatinine fails to achieve statistical or clinical reliability as an active diagnostic biomarker for thyroid disorders.

In summary, these aligned findings confirm that serum uric acid is a highly reliable, responsive indicator of thyroid-induced metabolic shifts. Routine screening and clinical tracking of serum uric acid are strongly recommended for newly diagnosed thyroid dysfunction cases to capture early metabolic imbalances and optimize patient care profiles [1,14].

This study has certain limitations. The cross-sectional design precludes establishing a causal relationship between thyroid dysfunction and alterations in serum uric acid or creatinine. The study was conducted at a single tertiary care center with a relatively modest sample size, which may limit the generalizability of the findings. In addition, potential confounding factors such as dietary purine intake, body mass index, physical activity, and other metabolic factors were not extensively evaluated. Further multicenter, longitudinal studies with larger sample sizes and adjustment for potential confounders are warranted to validate these findings.

## Conclusions

This study provides conclusive evidence that serum uric acid levels are significantly altered in patients with overt thyroid dysfunction, showing a strong positive correlation with serum TSH. Serum uric acid demonstrates fair and statistically significant diagnostic accuracy (AUC = 0.642, p < 0.001), with a threshold of ≥4.05 mg/dL serving as a highly sensitive clinical screening marker. In contrast, serum creatinine exhibits a significant negative correlation with TSH and is significantly lower in thyroid disease states but lacks the discriminatory power to serve as a reliable diagnostic marker (AUC = 0.441, p = 0.076). Consequently, routine screening of serum uric acid is highly recommended in newly diagnosed patients with thyroid dysfunction to systematically map and manage associated metabolic profiles.
